# Integration of mRNP formation and export

**DOI:** 10.1007/s00018-017-2503-3

**Published:** 2017-03-17

**Authors:** Petra Björk, Lars Wieslander

**Affiliations:** Department of Molecular Biosciences, The Wenner-Gren Institute, Stockholm University, 106 91 Stockholm, Sweden

**Keywords:** Gene expression, Splicing, Polyadenylation, Cell nucleus, Nucleocytoplasmic export, Export receptors, mRNA, Pre-mRNA processing, mRNP assembly, Nuclear pore complex

## Abstract

Expression of protein-coding genes in eukaryotes relies on the coordinated action of many sophisticated molecular machineries. Transcription produces precursor mRNAs (pre-mRNAs) and the active gene provides an environment in which the pre-mRNAs are processed, folded, and assembled into RNA–protein (RNP) complexes. The dynamic pre-mRNPs incorporate the growing transcript, proteins, and the processing machineries, as well as the specific protein marks left after processing that are essential for export and the cytoplasmic fate of the mRNPs. After release from the gene, the mRNPs move by diffusion within the interchromatin compartment, making up pools of mRNPs. Here, splicing and polyadenylation can be completed and the mRNPs recruit the major export receptor NXF1. Export competent mRNPs interact with the nuclear pore complex, leading to export, concomitant with compositional and conformational changes of the mRNPs. We summarize the integrated nuclear processes involved in the formation and export of mRNPs.

## Introduction

The product of a protein-coding gene that is delivered to the cytoplasm is an export competent mRNA–protein (mRNP) complex [1]. An mRNP contains a processed mRNA and a large number of associated proteins [2, 3]. The formation of an mRNP requires multiple and coordinated processes. The active gene is a crucial nuclear subdomain where synthesis, processing, and assembly of pre-mRNPs and mRNPs take place [4]. Chromatin structure, RNA polymerase II (RNA pol II) transcription elongation rate, and the integrated action of a number of processing machineries will all have an impact on the formation of the pre-mRNPs and mRNPs. Pre-mRNAs exist, very rarely, if ever, as complete copies of the gene, because the processing machineries that execute capping, splicing, and 3′ cleavage and polyadenylation also operate at the transcribing gene. Transcription by the RNA pol II elongation complex results in a pre-mRNA. During ongoing transcription, various proteins associate rapidly with the newly synthesized pre-mRNA. Furthermore, as a consequence of processing, defined proteins will be deposited onto the pre-mRNA at specific positions [5–8]. Proteins bind to pre-mRNAs in a highly ordered assembly process. The resulting pre-mRNPs change their structure as additional RNA is added, as additional proteins associate and as processing modifies the RNA. The associated proteins are important for every aspect of the fate of an mRNP, including export, localization in the cytoplasm, engagement with the translation machinery, quality control, stability, and degradation.

After release from the gene, an mRNP enters the interchromatin compartment and moves by diffusion mainly through the interchromatin channel network of diploid cells [9]. Some processing and protein acquisition can occur in the interchromatin, but to what extent an mRNP is modified, as to composition and structure remain to be determined. Apparently, mRNPs spend some time in the interchromatin. Varying sizes of pools of gene-specific mRNPs, therefore, exist. Unproductive encounters between the nuclear pore complexes (NPCs) and individual mRNPs can occur [10–12], either because the mRNPs do not have the necessary components, in this sense, are not export competent, or because the docking process at the NPC simply fails. When export competent mRNPs successfully dock at the NPCs, they are subsequently translocated through the NPC channel and thereafter released into the cytoplasm. During translocation, both conformational and compositional changes take place within the mRNPs.

**Fig. 1:**
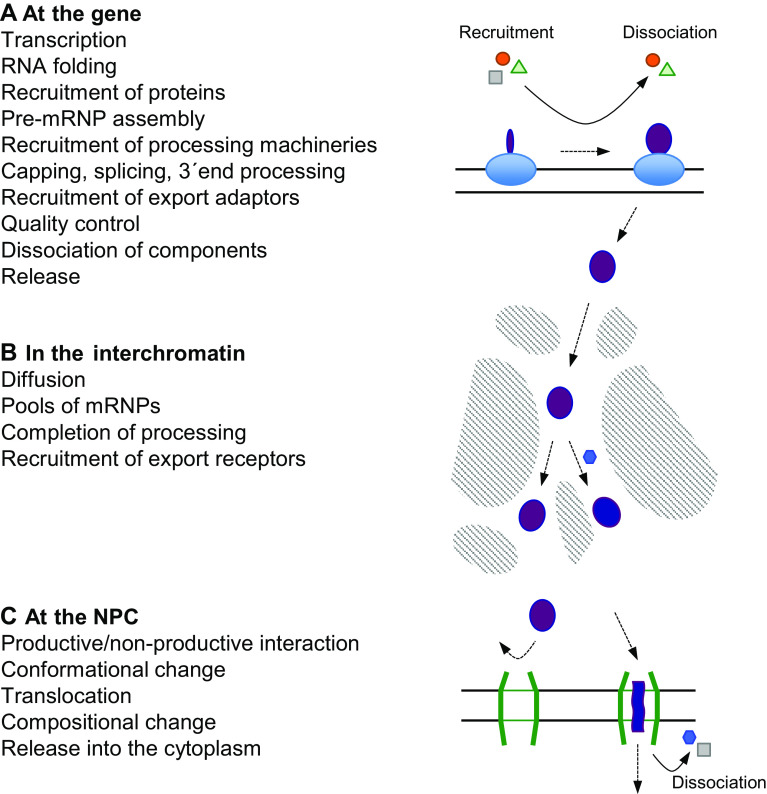
Schematic representation of the nuclear steps in gene expression. **a** At the gene. **b** In the interchromatin. **c** At the NPC. Processes that occur at each location are listed to the *left*. **a** The actively transcribing protein-coding gene (*parallel black lines*) provides an optimal environment in which RNA pol II (*blue oval*) produces a pre-mRNA (*purple oval*). During transcription, the pre-mRNA is folded and assembled with proteins (*green triangle* and *grey square*) into a pre-mRNP (*purple oval*). Several processing machineries (*orange circle*) transiently interact with the pre-mRNA, modify it, and leave protein marks at specific positions. **b** After release from the gene, processing can be completed and the mRNPs (*purple ovals*) move by diffusion within the interchromatin channel network, making up pools of gene-specific mRNPs. Additional components, for example the main export receptor NXF1, can be recruited to the mRNPs, that then become export competent (*dark blue oval*). Chromatin is depicted as striped areas. **c** mRNPs interact with the NPCs (*green*) that are imbedded in the nuclear membrane (*parallel black lines*). A majority of the interactions are non-productive and the mRNPs return into the interchromatin. If fully export competent, the mRNPs are translocated through the central channel of the NPC. The export is coupled to conformational and compositional changes of the mRNPs

We will outline the processes at the active gene, in the interchromatin and at the NPC that together result in the formation and export of functional mRNPs. It is clear that there is a close physical and functional coupling between transcription, processing, and assembly of pre-mRNPs and mRNPs. The mRNP obtains a processed structure and a collection of proteins that will influence the downstream events. The intricate physical and functional couplings are likely to have evolved, because they enhance coordination, efficiency, and regulation of the multiple steps of gene expression (Fig. 1). We will provide a cell biological view, focusing on the spatial and temporal aspects of formation and export of mRNPs, a perspective that is required for a full understanding of the intranuclear steps of gene expression. This cell biological view is largely based on studies of specialized, polytene cells that allow morphological, spatial, and temporal insights into the nuclear processes [13].

## Active genes—optimal nuclear microenvironments for transcription, pre-mRNP assembly, and processing

The cell nucleus contains several functional compartments which are maintained despite the lack of an enclosing membrane and the dynamic exchange of components with the surrounding nucleoplasm [14]. Compartmentalization creates microenvironments that are believed to facilitate coordination, efficiency, and regulation. The molecular mechanisms for establishing nuclear compartments are not well understood, but it has been suggested that they can be formed by either random self-organization or by an ordered assembly pathway. In addition, a so-called seeding mechanism could contribute [15, 16]. Several compartments form at sites of transcription, in a transcription-dependent manner, suggesting a role for RNA. The RNA can then nucleate the formation of the compartment. Both coding and non-coding transcripts are capable of attracting and retaining freely diffusible components from the nucleoplasmic pool [16, 17]. In vitro, interactions between RNAs and proteins can result in reversible membrane-free structures [18]. If such structures, so-called droplets or hydrogels, reflect the in vivo situation, special physical and biochemical properties could possibly favour different biological processes. RNA–protein assemblies may associate with components that play important roles in gene expression [19] or influence aggregates involved in diseases [20].

Transcribing genes may represent transient microenvironments favourable for mRNP formation (Fig. 1). Upon gene activation, the chromatin is extensively modified and unfolded, forming loops. Such loops can be readily visualized in specialized cells, such as amphibian oocytes [21] and polytene cells [13, 22]. Electron microscopy (EM) has revealed unfolded active genes also in mammalian diploid cell nuclei in perichromatin regions [23]. In situ hybridization experiments have also demonstrated unfolding of active genes [24]. Transcription activation can lead to transient physical proximity of the genes [25]. It has been suggested that transcription occurs in statically assembled structures, so-called transcription factories [26]. However, clustering of RNA pol II upon gene activation has been demonstrated to be transient [27]. In the polytene Balbiani ring (BR) gene loci, efficient recruitment of RNA pol II and proteins needed for pre-mRNP assembly and processing takes place upon transcription activation and apparently at the level of the individual gene locus. The presence of many active gene copies in the polytene chromosome results in a high local concentration of transcription and processing machineries that could be beneficial.

The functional significance of chromatin unfolding and the establishment of a microenvironment for transcription and pre-mRNA processing need to be further investigated. The relative contribution of chromatin modifications, the transcription process itself, and the presence of transcripts must be clarified. We need specific examples of endogenous genes in living cells to learn the rules and variations that appear to exist in different types of cells (mammalian diploid cells, yeast cells, polytene cells). Even if technologies have been developed, such as 3C (chromatin conformation capture), 4C-seq [28], and sophisticated fluorescence microscopy techniques, new methods are needed that allow analyses in vivo.

**Fig. 2:**
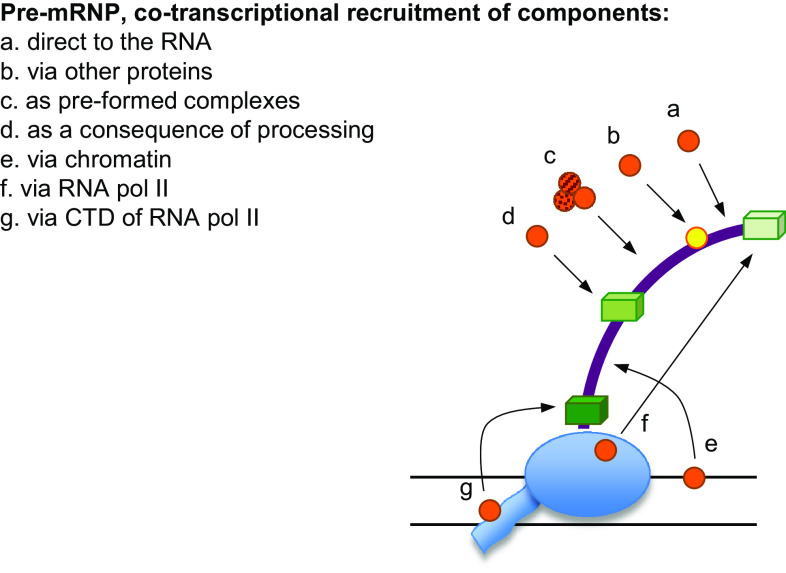
Recruitment of components for pre-mRNA processing and pre-mRNP assembly at the gene. The transcribing protein-coding gene (*parallel black lines*), the RNA pol II with its CTD (*blue*), the growing pre-mRNA (*purple*), processing machineries (*boxes* in *shades* of *green*), and various components (*orange circles*) are shown schematically. **a**–**g** Different pathways for recruitment of factors to the growing pre-mRNP. These pathways are briefly explained in the figure (listed to the *left*)

It is still not clear how recruitment of factors to active genes is brought about, although several mechanisms have been proposed (Fig. 2). Many proteins and preformed complexes, for example SR proteins and snRNPs, have affinity for the nascent transcript. The binding is sometimes mediated by specific sequences in the RNA [29–31]. Other proteins and complexes bind not directly to the transcript, but in layers of interactions to already bound components [32, 33]. While it is clear that protein modifications influence binding affinities, for example the phosphorylation status of SR proteins [34], it remains to understand to what extent diffusion and regulated affinities contribute. Other mechanisms could be important, such as preformation of sub complexes [35] and local recycling. The latter has been suggested for RNA pol II through coordination between transcription and 3′ end processing [36, 37]. Another way of coordinating interactions between the transcript and specific proteins is via the RNA pol II elongation complex. As the 5′ end of the RNA emerges from the exit tunnel, the capping enzyme, bound to the RNA pol II, is in position to rapidly cap the 5′ end [38]. The C-terminal domain (CTD) of the largest subunit of RNA pol II is implicated as a flexible interaction platform that helps to recruit different factors [39]. The CTD consists of many heptapeptide repeats that are phosphorylated differently during initiation, elongation, and termination of transcription. It has been shown that the RNA guanyltransferase binds to the CTD during transcription initiation [40, 41]. Several 3′ processing factors bind to the CTD [42–44], as well as the Transcription-Export (TREX) sub complex THO [45]. It is less clear to what extent spliceosomal components reach the nascent transcript via the CTD. The splicing factors U2AF65 [46] and PSF and its related protein p54^nrb^/NonO [47, 48] have been shown to bind to the CTD. Presumably, the role of the CTD modifications for recruitment of processing components will be even more appreciated. Methods to couple phospho-specific CTD modifications to interactions with defined proteins using specific antibodies combined with mass spectrometry is a promising start [49].

Structural studies of purified spliceosomes are improving [50], but high-resolution structural analyses of coupled transcription complexes and spliceosomes, at genes, are not yet reached [51]. The elongating RNA pol II contains additional components that help in recruiting mRNP biogenesis factors. In yeast, phosphorylated hexa-repeats in the elongation factor Spt5 are involved in recruiting capping enzymes [52], the 3′ end cleavage factor CFI [53], and the Paf1 complex [54] which is possibly involved in 3′ end processing.

## Influences of chromatin on transcription and pre-mRNA processing

The study of mRNP formation must consider all the coordinated events and interacting molecules at the active gene. One such consideration is the influence of chromatin on the transcription rate and pre-mRNA splicing [55]. The transcription rate can be uniform along exons and introns in long human genes [56], but it is also established that transcription rates can change along a gene [57, 58] and that this influences splice site choices [59]. The presence of alternative binding sites in the nascent transcript can lead to competition for binding of the interacting machineries. To what extent a high transcription rate results in the presence of alternative binding sites is likely to depend on the exon–intron structure, the binding affinities, and the kinetics of processing reactions. Therefore, a simple relationship does not exist between transcription rate and for example alternative splice site choices [60].

Nucleosome density and deposition of histone variants can modulate the movement of RNA pol II [61]. Nucleosomes are unequally distributed in exons and introns. In general, they are more closely packed in exons than in introns and may thereby contribute to different transcriptional elongation rates in introns and exons, respectively [62–65]. RNA pol II pause sites correlate with splice sites [66, 67]. Nucleosome positioning and composition could influence splice site recognition, although it is unclear to what extent the splicing process itself contributes to RNA pol II elongation slow down [68]. Several observations show that splicing and 3′ end processing can influence transcription [69–71].

A number of histone modifications have been shown to influence splicing. Some of these may work through affecting the RNA pol II transcription rate and others by influencing the recruitment of splicing factors. One example is the H3K36 methylation that plays a role in nucleosome positioning and in limiting elongation rate and thereby influencing splicing [72]. H3K36 methylation can also indirectly recruit the splicing regulators PTB and SRSF1 to the gene via so-called histone mark readers [73, 74]. In addition, the recruitment of spliceosomal components can be controlled by histone modifications [75]. In addition to methylation, other modifications of histones have been described, for example phosphorylation, sumoylation, and ubiquitination. It remains to be determined if also these modifications have an impact on splicing. An emerging field is the role of small and long non-coding RNAs in local alterations of chromatin modifications that will influence the splicing pattern [76].

The close contact between the chromatin and the pre-mRNP enables functional interactions between them. It has been reported that a pre-mRNP can influence transcription by recruiting a histone acetyltransferase to the active gene [77].

Chromatin structure is likely to affect transcription termination. Nucleosomes are enriched downstream polyadenylation sites [64], and may thereby be involved in RNA pol II pausing in the termination region. A possible mechanism could be R-loop induced antisense transcription leading to histone H3K9me2 modification and recruitment of heterochromatin protein1γ [78].

## Nascent pre-mRNP formation

### Pre-mRNA folding

All RNAs, including pre-mRNA and mRNA, have the ability to fold in alternative ways, following energy rules. Folding starts during transcription [79, 80]. The folding of an RNA is functionally important and it can influence interactions with trans-acting factors and itself be influenced by such interactions. Folding can have an impact on transcription [81] and the transcription rate can have an effect on RNA folding [82]. In addition, the folding of the pre-mRNA is dynamic, for example dictated by interactions with spliceosomes and not surprisingly, and the local structure in the pre-mRNA can affect splicing [83, 84]. Throughout the life of a pre-mRNA and an mRNA, the RNA exists in the context of an RNA–protein complex and this complex is repeatedly remodelled. Association of the pre-mRNA and mRNA with proteins has a profound effect on the folding of the RNA–protein complex.

### Proteins that interact with pre-mRNA and mRNA

The repertoire of proteins that associate with RNA is substantial and diverse [85]. Large-scale methods have broaden the identification of RNA–protein interactions, but in many cases, the functional importance of the interactions remains to be determined [29]. In general, RNA-binding proteins associate with RNAs using specific domains [30]. Several such domains have been characterized, for example the RBD (also called RRM) and the KH (hnRNP K-homology) domains. Many proteins with regulatory functions in gene expression contain multiple KH domains [86]. Other proteins involved in processes in gene expression contain one or more copies of RBDs. In fact, about 2% of the mammalian proteins contain this domain [87]. Examples include SR proteins [88], hnRNP proteins [89], poly(A)-binding protein (PABP) [90], and the 3′-processing protein CstF-64 [91]. RBDs have a typical structure and recognize crucial nucleotides in short degenerate sequence motifs. Sequence specificity and the binding domain sequence are often conserved [92]. In vitro, the binding affinity for individual tested domains is usually low, but in the cell, affinity might be higher, because other regions of the protein or even coupling to other proteins assist. In a given protein, an RBD can be combined with other kinds of domains that perform other types of functions, for example helicase activity or mediating protein–protein contacts. Proteins can recognize common mRNA structural elements, for example the m^7^G cap [93], the poly(A) tail [8], and the sugar-phosphate backbone [32]. Some proteins recognize specific sequence motifs, for example the poly(A) signal, and others associate in a sequence-independent manner with either secondary or tertiary folds [31]. They can be stably bound, for example as architectural elements that help to define mRNP organization, or transiently bound, for example involved in modulating specific steps of gene expression. Proteins can also bind indirectly to the pre-mRNA and mRNA via other factors, as in the exon junction complex (EJC) [32]. It should be pointed out that pre-mRNA processing machineries operate in the context of RNA–protein complexes and many proteins bind to the pre-mRNPs and mRNPs as a consequence of pre-mRNA processing, and examples include the cap-binding complex (CBC), the EJC, and PABPN1.

Approximately, 20 different proteins (named A1 to U) belong to the group of heterogeneous nuclear proteins (hnRNPs) [94, 95]. These proteins bind to many different RNA pol II produced transcripts. They can bind at several positions in the pre-mRNA with some sequence preference. The hnRNPs are preferentially present in cell nuclei. This reflects that they can leave the mRNP before export or that they rapidly shuttle back from the cytoplasm to the nucleus or a combination of the two. Some hnRNPs can accompany the mRNP to the cytoplasm and into polysomes. When bound to pre-mRNPs, they can affect packaging and stability. It has been suggested that hnRNP C tetramers organize the pre-mRNA [96]. The hnRNPs influence the transcripts in many different ways, for example as part of the pre-mRNPs, they can influence processing [97] and in the cytoplasm, they play roles in mRNP translation and stability [95].

Methodological improvements are needed to characterize the complete protein content of specific endogenous pre-mRNPs. One must consider that the composition of pre-mRNPs is dynamic over time and along a gene. The BR pre-mRNPs at the gene, and the BR mRNPs in both the interchromatin and at the NPCs, are morphologically identifiable. This fact has made it possible to decide, using immune-EM, where proteins associate with and dissociate from these specific endogenous transcripts. So far, 34 different proteins were identified within BR pre-mRNPs. These proteins have functions related to transcription, capping, 3´ end cleavage and processing, splicing, packaging, export, and quality control [13]. Some proteins, such as hrp36 (an hnRNP), a Y-box protein, and some SR proteins, become incorporated into BR pre-mRNPs and stay associated with the mRNPs throughout export and into polysomes. Some proteins like Rrp6, UAP56, ALY/REF, RSF, and PABPN1 associate with the BR pre-mRNPs and leave the BR mRNPs at the NPC.

### Pre-mRNP assembly and packaging

During transcription, the growing pre-mRNA is rapidly and continuously assembled into a pre-mRNP complex, probably as soon as the transcript emerges at the surface of RNA pol II [98]. Many different proteins and processing machineries are incorporated (Fig. 2) [22, 99], but the mechanisms of recruitment are not yet fully elucidated. Some of the interacting components bind to essentially all pre-mRNAs, such as the capping enzymes, CBC proteins, and PABPN1. The protein content in pre-mRNPs is also partly gene-specific, depending on the sequence and exon–intron structure of the pre-mRNA. The pre-mRNPs from different genes contain different combinations of proteins. This is exemplified by the observation that pre-mRNPs from different genes contain different combinations of members of the SR protein family. The protein distribution within pre-mRNPs also differs between genes [100, 101].

The molecular background for packaging of pre-mRNPs is unknown in almost all cases. It has been suggested that interactions between SR proteins and the EJC compact pre-mRNPs [102]. Dimerization of the poly(A)-binding protein Nab2, not only influences poly(A) tail length, but may also contribute to mRNP compaction [103]. Data show that specific pre-mRNPs are packaged into compact RNA–protein complexes, in close proximity to the elongating RNA pol II. High-resolution imaging of lampbrush nascent transcripts shows that these transcripts are tightly packed with proteins [104].

In perichromatin regions in mammalian cell nuclei, short fibrillar and granular structures presumably represent nascent pre-mRNPs [105]. The packaging of the endogenous BR1 and BR2 pre-mRNPs has been described using EM [22]. These approximately 40 kb-long pre-mRNAs, consisting mainly of exon sequences, are initially packaged into thin, 5–10 nm pre-mRNP fibers. Remodelling takes place, resulting in an approximately 20 nm thick, flexible RNP in which a thin 7 nm fibre probably is a basic structural element. The 20 nm fibre grows at its root until obtaining a length of 90 nm. At this stage, about 8 kb of the transcript is incorporated. Then, a conformational change at the tip of the 20 nm fibre results in a ribbon with a diameter of 26 nm, and subsequently, this ribbon compacts into a ring-like RNP with a final diameter of 50 nm. In contrast, in the BR3 gene, the pre-mRNA contains alternating short exons and introns throughout its length. The BR3 gene is 10.5 kb long, but because of the structurally dynamic splicing process, a full-length pre-mRNA is not present. The BR3 pre-mRNP structure is then dominated by the assembly, action, and release of spliceosomes [51]. Evidently, the exon–intron organization greatly influences the packaging of the pre-mRNPs. So far, a completely processed BR3 mRNP has not been possible to identify, and therefore, no structural information is available.

The evolution of packaging of the pre-mRNP and mRNP has presumably been important for a number of reasons. Packaging might reduce the formation of RNA–DNA hybrids, so-called R-loops, which can cause genomic instability [106]. Furthermore, packaging of the pre-mRNP and mRNP in compact complexes probably facilitates diffusion through the interchromatin and at the same time provides a stable but yet flexible complex that can change structure and composition at the NPC.

## Pre-mRNP processing at the gene and coupling to export

At the gene, the pre-mRNA interacts with the processing machineries responsible for capping, splicing, and 3′ cleavage and polyadenylation. The processing machineries all contribute to the composition and structure of the pre-mRNP and all leave marks in the mRNP that are important for subsequent events in gene expression, including export.

### Capping

Capping enzymes recognize the 5′ end of the pre-mRNA, essentially as soon as it appears at the RNA pol II exit tunnel [38]. The nuclear cap-binding complex proteins CBC20 and CBC80 then bind to the cap, forming the CBC, also at the gene [107]. The CBP80 protein is important for recruitment of the TREX complex, including the export adaptor ALY/REF [108]. In the cytoplasm, the CBC is involved in the initial translation and is then replaced by eIF4E for efficient translation [5].

### Splicing

Intron excision requires the multicomponent spliceosome [109]. Spliceosome assembly takes place during ongoing transcription [110, 111], evidently for a majority of pre-mRNAs [112–116]. Close proximity between the spliceosome, the RNA pol II, and the chromatin has been demonstrated [51]. Many studies have reported interactions between individual components of transcription and processing machineries. In most cases, these interactions, and especially their functional significance, are difficult to evaluate. We need improved methods to characterize and functionally evaluate protein–RNA and protein–protein interactions at active genes in living cells. In vivo rates for the splicing process have been reported to range from 30 s to several minutes [117–119]. Recent studies have demonstrated that the spliceosome can assemble on the pre-mRNA already when the RNA pol II has synthesized in the order of 24 nucleotides downstream a 3′ splice site [120]. Efficient RNAseq methods combined with robust extraction of nascent transcripts are required for analysis of additional endogenous genes to learn the rules. Understanding the principles for constitutive splicing will be important for understanding regulation of splicing. Measurements of the precise kinetics of alternative splicing will be important to verify the molecular mechanisms involved. It will also be important to learn if and to what extent the rate of splicing can be modified. Introns are excised in an overall 5′ to 3′ order [117, 121], but all introns are not necessarily excised at the gene. In a population of gene-specific pre-mRNAs, some of the transcripts lose their introns in the interchromatin [111, 121]. This may be due to variation in splicing kinetics, the length of the gene [56, 111], and alternative splicing regulation. A nascent pre-mRNP can contain more than one intron, showing that spliceosome assembly and activity do not stall the RNA pol II until an intron is excised [51].

SR proteins are characterized by having one or more RNA-binding domains and a domain rich in serine-arginine repeats [122]. SR proteins bind to many different pre-mRNAs in gene-specific combinations [100, 101]. SR protein binding is at least partly sequence specific and is essential for the splicing reaction. Their phosphorylation level is important for the recruitment to pre-mRNAs and for the splicing reaction. SR proteins are required for constitutive splicing and can influence alternative splice site choices [123]. SR proteins influence also other steps in the biogenesis of mRNPs [124, 125]. As pointed out, SR proteins, in combination with EJC core components, may compact mRNPs [102]. SR proteins remain associated with mRNPs and are important for export, serving as export adaptors for NXF1, the main mRNP export receptor. It has also been described that SR proteins regulate 3′ processing of pre-mRNAs [126], mRNA stability [127], and translation initiation [128].

Splicing is not only necessary for obtaining correct open reading frames. Splicing also leads to deposition of the EJC core [129]. EJCs associate in a splicing-dependent manner with pre-mRNAs, 20–24 nucleotides upstream the boundary between two joined exons [6]. The EJC core contains four proteins, eIF4AIII, the heterodimer Mago-Y14 and Barentsz. All four core proteins associate with the nascent pre-mRNA [130]. Mapping of binding sites shows that the full set of EJC core proteins, including Barentsz, are present at most exon–exon junctions, but additional binding sites exist for eIF4AIII [102, 131, 132]. The EJC core has the ability to recruit different proteins and thereby plays central roles in several post-transcriptional processes [129, 133]. These include recruitment of export adaptors and translation initiation factors and assembly of a functional nonsense-mediated decay (NMD) complex.

### 3′ end cleavage, polyadenylation, and termination of transcription

Essentially, all pre-mRNAs are cleaved and polyadenylated by the 3′ cleavage and polyadenylation machinery. The processes and responsible components are biochemically well characterized. Proper 3′ end formation is necessary for mRNA export [134]. More than 70% of the mammalian protein-coding genes produce isoforms differing in alternative polyadenylation sites [135].

A part of the polyadenylation machinery is recruited to the transcript via the RNA pol II CTD [42, 43]. Interactions between the TREX components ALY/REF and THOC5 with several proteins of the 3′ processing machinery indicate that TREX is important for 3′ processing and possibly for release of the transcript from the gene. The cleavage reaction takes place at the gene. In the BR1 gene, microdissection revealed that a population of correctly spliced and cleaved mRNPs was present at the gene. This population had a short poly(A) tail, 10–20 adenylate residues long [136]. In the interchromatin, a switch from a distributive to a processive elongation mode of polyadenylation then took place, adding on average 100 adenylate residues. The presence of a population of mRNPs with short poly(A) tails at the gene could be due to retention of the mRNP during the initial polyadenylation reaction, possibly involving the 3′ processing machinery. Moreover, it could reflect the initial distributive phase of polyadenylation observed in vitro. The length of the poly(A) tail is controlled by PABPN1 [8]. Other proteins are also involved, for example the mammalian protein Nucleophosmin 1 that is deposited onto the mRNA upstream the poly(A) signal [137]. Release of mRNPs from the gene requires splicing, cleavage at a correct polyadenylation signal, and polyadenylation [138]. The mechanism for mRNP release from the gene and the potential relationship to termination of transcription is still unclear.

Some genes are extremely long, emphasising that synthesis of pre-mRNAs is dependent on the processivity of RNA Pol II [56]. Synthesis is also dependent on the recognition of appropriate transcription termination signals [139]. During transcription, RNA Pol II may be stalled or prematurely terminated [139]. Several situations can influence transcription termination, for example viral infection [140], cancer [141] and osmotic stress [142]. Transcription termination influences alternative poly(A) site usage [135]. This is functionally important, because mRNA isoforms with different lengths of their 3′ UTRs can have different stability, localization in the cytoplasm, and translation properties [143].

Studies in yeast show that both Nab2p and Pab1p, the mammalian orthologs PABPN1 and PABPC1, bind poly(A) tails. Nab2p is predominantly located in the nucleus and Pab1p in the cytoplasm. Nab2p protects poly(A) RNA from degradation by the exosome [144] and promotes export by interacting with the NPC protein Mlp1 [145]. Nab2p may further interact with the spliceosome and with Rrp6 to integrate splicing and pre-mRNP decay [146].

## Quality control

Synthesis and processing of pre-mRNPs are complex molecular events that are not always perfectly performed. Aberrant products can be made and the cell must be able to handle these. The presence of quality control systems has been demonstrated in cells having specific mutations in the RNA or by knocking down enzymes that normally degrade aberrant RNAs. Transcripts with defects in capping, assembly, splicing, and 3′ end formation have all been shown to be degraded [147]. Degradation can occur at the gene by 5′–3′ exonucleases such as Xrn2 or by the exosome [148].

Transcripts with retained introns or defective 3′ ends have been shown to be retained at the gene [149–152]. The exosome is needed for this retention [147]. In yeast, export-incompetent mRNPs are retained at the gene through the action of the chromatin remodelling complex ISW1 in cooperation with Rrp6 [153]. The retention can lead to degradation [154], or an alternative outcome could be increased time for completion of processing [155]. It is likely that the degradation, processing, and RNA–protein assembly events balance each other. These balances may be examples of kinetic proofreading [156].

In a broader sense, it can be argued that also the deposition of proteins in connection to pre-mRNA processing provides a quality control. For example, a non-correct EJC will not support downstream processes such as export and translation.

## Recruitment of export adaptors is linked to transcription and processing

**Fig. 3:**
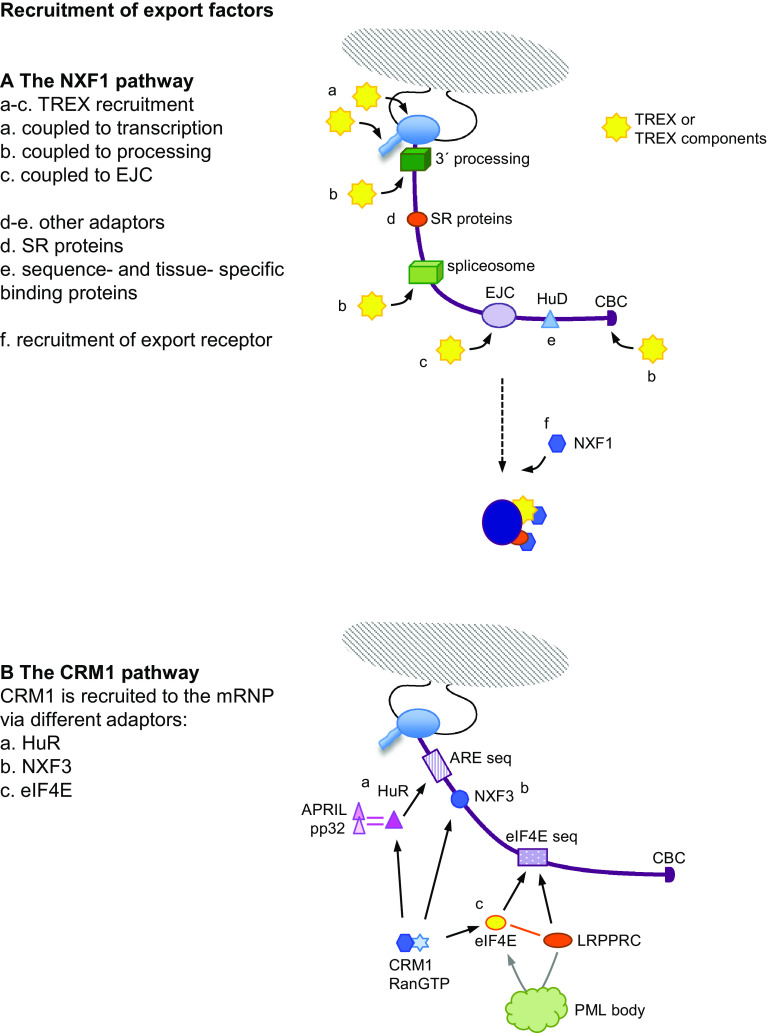
Export of mRNPs depends on several different export adaptors and two main export receptors. **a** NXF1 pathway. The transcribing gene (*black line*), the RNA pol II with its CTD (*blue*), processing machineries (*boxes* in *shades* of *green*), the EJC (*light purple oval*). **a**–**c** TREX or subcomponents of TREX (*yellow stars*), for example ALY/REF, THOC2 and THOC5 bind to the pre-mRNA (*purple line*) through several different pathways (listed to the *left*). (d) SR proteins (*orange oval*) can also serve as export adaptors to NXF1. **e** In neurons, the HuD adaptor protein (*blue triangle*) binds sequence specifically to the RNA. **f** NXF1 (*blue hexagon*) is subsequently recruited, via the export adaptors, to the mRNP, that becomes export competent (*dark blue oval*). Binding of export adaptors is shown to occur at the gene (as been shown to occur for some of the adaptors). Recruitment of NXF1 occurs in the interchromatin [121]. **b** CRM1 pathway. Export of a subset of mRNPs requires the export receptor CRM1. The transcribing gene (*black line*), the RNA pol II with its CTD (*blue*) and the pre-mRNP (*purple*). CRM1/Ran-GTP (*blue hexagon*/*blue star*) is recruited via several different adaptors, HuR (*pink triangle*), NXF3 (*blue circle*), and eIF4E (*yellow oval*) (**a**–**c**). HuR has two cofactors, April and pp32 (*light pink triangles*), and is recruited to ARE sequences (*striped box*). The protein LRPPRC (*orange oval*) promotes the release of eIF4E (*yellow oval*) from PML bodies (*green*) and the binding to a specific RNA eIF4E sequence (*dotted box*)

An mRNP released from its gene is in several aspects likely to be ready for export to the cytoplasm. It is folded in a compact but flexible manner. It is equipped for downstream processes, with a CBC, a large number of different proteins, a poly(A) tail, and it is spliced to have a translatable open reading frame. The question is what more an mRNP must contain to be export competent in the sense that it can be channelled through an NPC. An obvious answer is that each mRNP requires export adaptors and export receptors. Evolution has resulted in multiple different export adaptors and their recruitment to mRNPs is promoted by transcription and processing. The two main export receptors, NXF1 and CRM1, interact with different sets of export adaptors (Fig. 3). Many adaptors are present in the mRNP at the time of release from the gene.

The TREX complex is a conserved complex that has functions in transcription elongation, mRNA export, and genome stability [157, 158]. It includes the THO complex and the helicase UAP56. UAP56 promotes spliceosome assembly and is also an EJC component. In addition, many other proteins are found in the TREX complex [158], among these, the export adaptor ALY/REF. Recruitment of TREX to the pre-mRNP is coupled to transcription elongation, capping, splicing, and 3′ end processing. The recruitment is facilitated through the association with the Ser2 phosphorylated CTD of RNA pol II [45, 159] and by the elongation Prp19 complex [160]. TREX can be recruited to the 5′ UTR of the pre-mRNA, dependent on the CBP80 protein [108] and on splicing [161]. The TREX subunit ALY/REF interacts with the EJC component eIF4AIII [162], making it likely that the EJC is involved in loading TREX onto the pre-mRNA. In yeast, ALY/REF can also interact with Pcf1, a 3′ cleavage factor [163]. UAP56 is involved in recruiting ALY/REF [164] and other proteins that influence export adaptor function. The mammalian DDX39 protein is a UAP56 paralog and has overlapping functions. UAP56 has also been shown to interact with UIF, which can bind to NXF1 and thus serve as an export adaptor [165].

TREX-2 is another conserved protein complex that has been shown to interact with the NPC basket [166, 167] and also with the export receptor NXF1 in mammals and Mex67 in yeast [168, 169]. In addition, studies in yeast have shown that subunits of TREX-2 interact with the SAGA histone acetylase complex and the promoter-bound Mediator complex [170, 171] and could thereby provide a functional link, and possibly physical coupling, between transcription initiation and mRNP export through the NPC. The TREX-2 subunit GANP can interact directly with NXF1, but it is unclear whether it is a general export factor. It has been shown that GANP is involved in the selective export of specific mRNAs, including those coding for components involved in mRNA processing and ribosome biogenesis [172]. The GANP-dependent export pathway may thereby facilitate rapid changes in gene expression.

SR proteins can serve as export adaptors [173, 174]. SR proteins remain bound to mRNAs after splicing and different mRNPs have different combinations of SR proteins [100, 101]. SRSF3 has been shown to bind to the last exon and may promote export of mRNA isoforms with long 3′ UTRs, by recruiting NXF1 [175].

## mRNPs in the interchromatin compartment

**Fig. 4:**
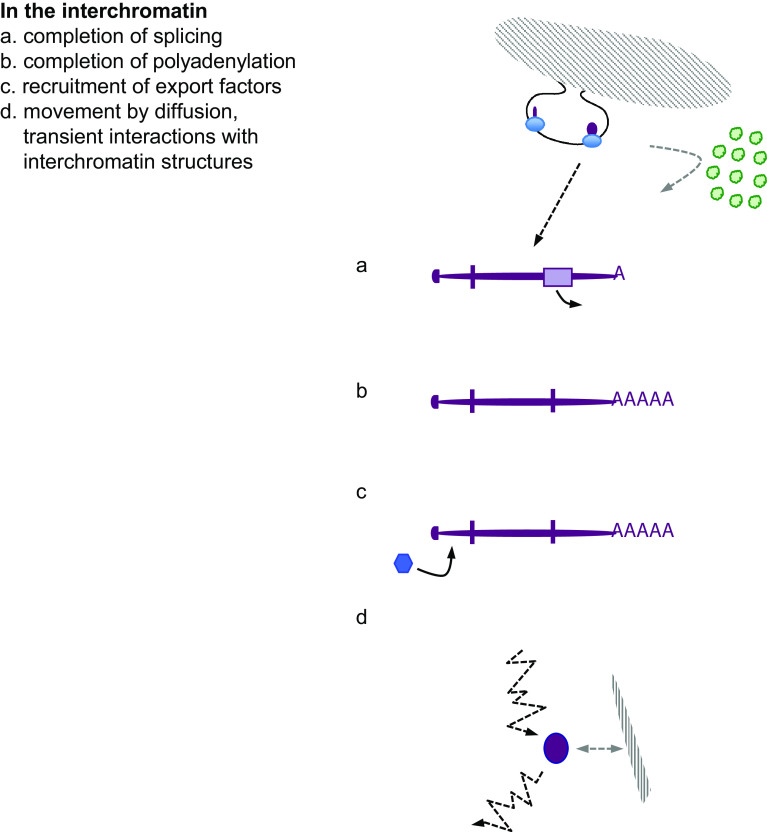
Processes taking place during mRNP transport through the interchromatin. An actively transcribed gene is shown as an unfolded loop (*black line*), with RNA pol II (*blue*) and pre-mRNPs (*purple*). **a** After release from the gene, splicing can be completed (intron, *purple box*), for some pre-mRNPs possibly involving interchromatin granule clusters (*green circles*). **b** Polyadenylation can be completed after release from the gene. **c** Export receptor NXF1 (*blue hexagon*) is recruited via its adaptors. **d** Folded mRNP (*purple oval*) may transiently interact with interchromatin structures (*grey striped*) during diffusion (*irregular lines*) within the interchromatin

Even if many processes in mRNP formation occur at the gene, important events such as completion of processing, binding of export receptors, and intranuclear transport take place in the interchromatin compartment (Fig. 4). The protein content of gene-specific mRNPs, and how this might change in the interchromatin, is essentially unknown. This is difficult to analyse, and so far, only average data for mRNP populations are available. The fact that most mRNPs cannot be structurally studied in diploid cells is further a drawback. Until better methods are developed, we rely on special cell types and special genes, such as polytene cells and BR mRNPs, for model studies.

### Processing in the interchromatin

Splicing can occur post-transcriptionally. Fractionation of cell nuclei, followed by quantification of the catalytically active SF3b155 protein, estimated that about 20% of splicing takes place in the nucleoplasmic fraction [112]. Several other examples of post-transcriptional removal of introns have been reported [83, 176–178]. The time remaining from the synthesis of an intron until transcription termination and 3′ processing influences if intron excision is completed co- or post-transcriptionally [56, 111, 121]. In a population of gene-specific pre-mRNAs, excision of a given intron can take place during transcription in most transcripts and be completed after release from the gene in the remaining transcripts [111]. This suggests that even if spliceosome assembly is initiated during transcription, the splicing reaction does not need to occur at the gene. An interesting question is, if co-transcriptional splicing requires the RNA Pol II and its CTD, how can post-transcriptional splicing do without it? Possibly, the initial steps of spliceosome assembly take place during transcription, while completion of splicing can be performed away from the gene.

Clusters of granules in the interchromatin, called interchromatin granule clusters at the EM level and speckles at the light microscope level, contain high concentrations of spliceosome components [179]. The full functional significance of this compartment remains to be elucidated. Storage sites for spliceosomal components are one possibility and it is well established that SR proteins are recruited from the granule clusters to nascent pre-mRNPs and that phosphorylation regulates this recruitment [180]. Transcribing genes are often located at the surface of the granule clusters. The proximity between active genes and the granule clusters may well-facilitate spliceosomal recruitment to the nascent transcripts. Active spliceosomes have been detected in speckles [112], suggesting that some introns are excised in this environment. During influenza A viral infection, a balance between synthesis of unspliced M1 mRNA and spliced M2 mRNA is established. The M2 mRNA seems to be generated by splicing within interchromatin granule clusters. It may be a way to ensure efficient supply of spliceosomal components. Interestingly, the viral NS1 protein in combination with a host NS1-binding protein is needed for targeting the M1 mRNAs to the speckles [181].

Synthesis of the poly(A) tail can be completed in the interchromatin. In BR genes, isolation by microdissection showed that polyadenylation is initiated at the gene and can be completed after release into the interchromatin [136].

### Structure of mRNPs

So far, we know very little about the structure of endogenous gene-specific mRNPs. The best characterized examples are the BR1 and BR2 mRNPs. These mRNPs, containing approximately 40 kb-long mRNAs, have a well-defined three-dimensional structure, and this structure has a hierarchy of folding that is established largely during transcription. At a first level, a 7 nm mRNP fibre is further folded, forming a 26 nm ribbon. This ribbon is folded into an overall ring-like complex [182].

In yeast, EM analyses of purified mRNP populations suggest that mRNPs have an extended shape with lateral restrictions [183]. The length of these ribbon-like structures increased when the length of the mRNA increased, while their diameter remained approximately 5 nm. The mRNA in mRNPs is thus folded into a compact structure. Furthermore, the length of the mRNA influences the overall dimensions of the compacted mRNPs.

### Association of proteins with mRNPs in the interchromatin

High sensitive mass spectrometry analyses of proteins that UV-crosslink to and co-purify with poly(A) RNAs have revealed a large number of proteins in direct contact with mRNAs. In mammalian cells, approximately 800 proteins are associated with the mRNA population [184, 185]. Enriched yeast mRNPs have been shown to contain several examples of export factors [183]. Purified in vitro spliced defined mRNPs contain approximately 45 different proteins [186].

Studies of endogenous gene-specific mRNPs are rare, but required for knowledge of the fate and behaviour of mRNPs. Experimental advantages make it possible to analyse properties of the BR1 and BR2 mRNPs. Already at release from the gene, these mRNPs are packaged and equipped with the majority of its associated proteins. BR1 and BR2 mRNPs have a protein content of about 60%, presumably corresponding to about 500 individual proteins [187]. Some proteins are likely to be present in multiple copies in a single mRNP. Immune-EM has identified 41 different proteins within BR pre-mRNPs and mRNPs. These proteins have functions related to transcription, capping, 3′ end cleavage and processing, splicing, packaging, export, quality control, and translation [13].

BR mRNPs retain their morphology at the ultrastructural level from the release from the gene until translocation through the NPC. Even so, immune-EM has demonstrated that BR mRNPs recruit some proteins in the interchromatin. One example is the RNA helicase Ct-hrp84 [188]. Most notably, this is the case for the export receptor NXF1 and the NMD factors UPF2 and UPF3 [13]. The BR pre-mRNPs and the mRNPs released from the gene contain several export adaptors and it is so far unknown why NXF1 is not recruited until the mRNP is present in the interchromatin. Possible explanations include necessary structural adaptor rearrangements in the BR mRNP and requirement of so far unknown factors only present in the interchromatin. In mammalian cells, NXF1 has been located throughout the nucleus but not at active genes [189]. Furthermore, NXF1 has been shown to interact with ALY/REF close to speckles [190].

### mRNA modifications

Modified bases are important for the function of non-coding RNAs, such as tRNAs and rRNAs. More recently, it has been discovered that also mRNAs are subjected to chemical modifications that can influence and regulate their function and fate [191–193]. So far, *N* ^6^-methyladenosine, pseudouridine, 5-methylcytosine, *N* ^1^-methyl6adenosine, and 5-hydroxymethylcytosine modifications of coding RNAs were identified in eukaryotic cells. The m^6^A modification is the one most thoroughly analysed and described to date. The modifications can induce conformational changes of the RNA. They can also interfere with RNA–protein interactions by either blocking or facilitating the binding of specific proteins.

The enzymes that mediate and the enzymes that remove the modifications are mostly found in the nucleus, but can, in some cases, also be localized to the cytoplasm. Thus, both nuclear and cytoplasmic pre-mRNAs and mRNAs can be targeted and, perhaps, influenced in different ways, depending on the location. Studies of the modifications identified so far have reported consequences for splicing, export, translation, and stability of the pre-mRNPs and mRNPs [194, 195].

### Export receptors

For export, mRNPs depend on association with export receptors (Fig. 3). These receptors mediate productive interactions with the NPC. Export receptors need adaptor proteins for binding to mRNPs. The majority of mRNPs uses the receptor NXF1/NXT1 heterodimer for their export [157, 196, 197]. NXF1 has an N-terminal RBD and can bind to RNA weakly, but with increased affinity in the presence of ALY/REF. It has a leucine-rich region that together with the RBD can bind to CTE motifs in viral RNAs [198]. In addition, NXF1 has a C-terminal ubiquitin-associated domain and a domain similar to that found in NTF2. This domain is important for several protein interactions [199]. The two latter domains can interact with FG-containing NPC proteins. NXF1 binds weakly and unspecific to mRNA, but upon recruitment via the adapter ALY/REF and THOC5, NXF1 is remodelled to expose its RBD, facilitating the binding to RNA [200]. Consistent with NXF1 binding to several export adaptors, at the 5′ and 3′ ends, as well as within transcripts, it is likely that multiple copies of NXF1 bind to individual mRNPs. Analysis by iCLIP has demonstrated many NXF1-binding sites in close proximity to the SR protein SRSF3 [175]. NXF1 interacts with several different proteins of the NPC during the translocation process. This will be discussed below.

The major protein exporter CRM1, in association with Ran-GTP [201], is also required for export of rRNPs, snRNPs, microRNAs, and tRNAs. In addition, it serves as an export receptor for a subset of mRNPs. CRM1 binds indirectly to mRNPs via different adaptors. Some mRNAs contain AU-rich sequence elements (AREs) in their 3′ UTRs. The Human antigen R (HuR) protein that contains a Nuclear Export Signal (NES) binds to these elements and, in turn, binds CRM1. HuR has two ligands, pp32 and April, that link HuR to CRM1. The requirement for the HuR ligands appears to be mRNA specific. The ligands have been shown to mediate export of ARE-containing c-fos mRNA [202–204].

A number of mRNAs have a 50 nucleotides-long sequence in their 3′ UTR that is sufficient to direct these mRNAs into an eIF4E and CRM1-dependent export pathway [205]. This so-called eIF4E-sensitivity element binds the adaptor protein, LRPPRC. This protein relocates eIF4E from PML bodies, possibly by competing for overlapping binding sites on eIF4E and also promotes the association of eIF4E with mRNAs [206]. LRPPRC mediates contact with CRM1, allowing transport through the NPC.

The NXF family member NXF3 can also recruit CRM1 to mRNPs.

Several observations show that interactions between export adaptors and receptors are more diversified than previously thought. Binding of the main mRNP export receptor, NXF1, to export adaptors shows variation. For example, in addition to SR proteins and ALY/REF, the neuron-specific HuD protein is an adaptor for NXF1 [207]. Components of TREX, other than ALY/REF, can contribute in recruiting NXF1. For example, THOC2 and THOC5 are involved in export of subsets of mRNPs [208], and THOC5 is required for export of heat shock HSP70 mRNPs [209].

The combination of various ways of recruiting different export adaptors, dependent on processing and sequence specificity, and the subsequent binding of the export receptors, show that there are alternative export pathways. Through such diversified interactions, export of selected subpopulations of mRNAs can occur [210, 211]. During cellular stress, stress-specific mRNAs elude export quality control using an alternative mode of binding the export receptor Mex67 [212].

### Pools of mRNPs in the nucleus and movement through the interchromatin

Diploid cell nuclei contain chromosome territories and different compartments [213, 214]. Active chromatin is found in perichromatin regions at the periphery of the inactive chromatin. The interchromatin compartment makes up nearly 50% of the nuclear volume and forms an irregular network of narrow channels, often connected to NPCs. The differences in chromatin density are likely to considerably affect the movement of mRNPs.

EM analyses have demonstrated that BR mRNPs in polytene nuclei, largely devoid of chromatin, move in all directions after release from the genes [215]. This observation is in agreement with studies of single mRNPs in diploid cell nuclei where no directionality in mRNP movement has been recorded. Accordingly, inside nuclei, mRNPs move by diffusion [216–219]. The movement is likely to be restricted by the chromatin and mainly taking place through the network of interchromatin channels. It has been calculated that the movement of mRNPs from the active gene to the NPC takes between 6 and 50 min in diploid nuclei [9, 220]. Different diffusion characteristics have been described for different mRNPs. Small mRNPs tend to move faster than large mRNPs [9]. Thus, a nucleus contains pools of mRNPs and the pools are of different sizes for specific mRNPs. For a specific gene, the size of such a pool is likely to be influenced by transcription, processing, and diffusion characteristics. Nuclear pools of processed mRNPs have been described for several genes and suggested to buffer the level of cytoplasmic mRNPs [221].

Quantitative measurements and calculations for BR mRNPs show that the interchromatin in polytene nuclei contains a pool of BR mRNPs. No or little degradation of BR mRNPs takes place in the nucleus [222]. In vivo, individual BR mRNPs in the pool move irregularly by diffusion and they interact occasionally with interchromatin fibers [223–225]. Approximately equal numbers of BR mRNPs, about half the size of the interchromatin pool, are synthesized and are exported to the cytoplasm per time unit. Immune-EM analyses show that BR mRNPs associate with the export receptor CRM1 at the gene [226], although CRM1 does not seem to be important for export of BR mRNPs. The BR mRNPs also associate with several NXF1 adaptors, for example SR proteins. However, in the interchromatin pool, only about 25% of the BR mRNPs contain NXF1 at a given moment [130]. It is, therefore, possible that BR mRNPs move around by diffusion in the interchromatin and randomly bind NXF1.

In the nuclei of mammalian cells, it appears that pools of poly(A) RNAs with retained introns are frequent [227, 228]. It has also been shown that subsets of introns in mammalian mRNAs have long half-lives and that their excision is influenced by the kinase Clk, possibly by affecting the phosphorylation of SR proteins and splicing [228]. Export of unspliced mRNAs may normally be restricted [229, 230]. Restricted export of incompletely spliced mRNAs using NXF1 involves the NPC associated protein Tpr [231]. However, both endogenous and viral mRNAs with retained introns can be exported and translated [232–234]. Incompletely spliced mRNAs are often subjected to degradation by the NMD pathway.

It is likely that a balance between intranuclear processing, degradation, and diffusion regulates mRNP export and thus influences the size of the pool of specific mRNPs. Retention and degradation of incompletely spliced mRNAs may be involved in neuronal development. It has been proposed that the protein PTB represses splicing, and that nuclear degradation of the incompletely spliced mRNA follows. The exosome and Tpr are involved [235]. Specific cases of intron retention may be important in homeostatic control of gene expression. The PABPN1 is such an example, where an interplay between the level of PABPN1, retention of a 3′ terminal intron, and nuclear exosome activity regulates PABPN1 synthesis [236].

## At the nuclear pore complex (NPC)

The NPC mediates almost all bidirectional transport of molecules between the nucleus and cytoplasm. In special circumstances, unconventional transport pathways may be used. For example, viruses can rebuild the NPC, allowing transport of viral mRNPs [237] and large mRNPs can exit the nucleus through nuclear envelope budding [238, 239].

**Fig. 5:**
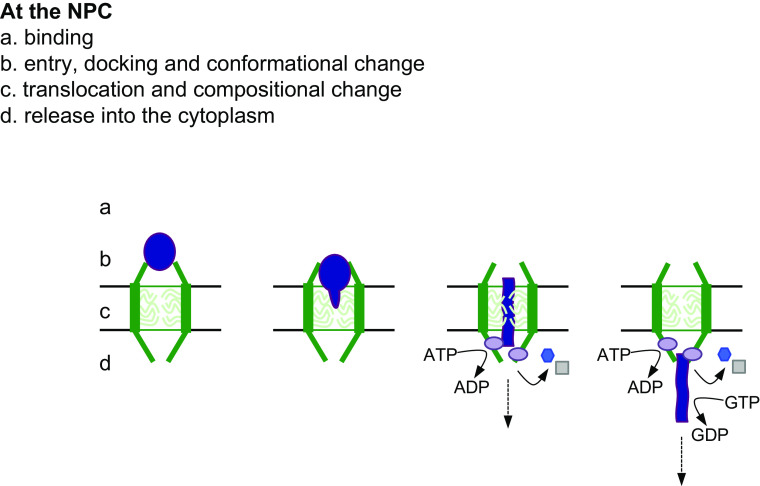
Principal steps during mRNP transport through NPCs (*green*). The *parallel black lines* indicate the nuclear membrane. **a** The export competent mRNP (*dark blue*) interacts with the basket of the NPC (*green*). **b** mRNP changes conformation and is fed into the channel of the NPC, where interaction with FG-repeats (*light green*) occur. **c, d** mRNP changes conformation extensively in the central channel during translocation. The cytoplasmic fibers are in close contact with the exit of the channel. Here, the mRNP looses many, but not all proteins (*blue hexagon* and *grey square*). The helicase Dbp5 (*light purple ovals*) associated with the fibers and ATP hydrolysis are involved. In the CRM1-dependent export pathway, GTP hydrolysis is required for mRNP release

As shown in the BR system, an mRNP is largely produced at the gene and appears only modestly reorganized or modified in the interchromatin compartment [13]. Additional studies of specific mRNPs are needed to substantiate this conclusion. In contrast, during interaction of the mRNP with the NPC, drastic structural and compositional changes take place, concomitant with its delivery into the cytoplasm (Fig. 5).

### Structure and composition of NPCs

The structure and composition of NPCs are well characterized [240, 241]. The size of an NPC is estimated to be between 50 and 125 MDa, depending on the species. The NPC has an eightfold ring symmetry. The core consists of a central channel positioned between a nuclear and a cytoplasmic ring. Fibrillar structures are present on both the nuclear and cytoplasmic sides. An NPC is built from about 500–1000 individual proteins belonging to the approximately 30 different types of nucleoporins (Nups). The transmembrane Nups anchor the NPC in the nuclear membrane. The structural Nups provide a scaffold for other Nups. The FG Nups contain domains with repetitive motifs of phenylalanine (F) and glycine (G) residues. The FG-rich domains are flexible and disordered, and they fill the central channel of the NPC, forming a barrier. Small molecules can diffuse through the NPC, but as the size reaches approximately 5 nm in diameter and/or 30–40 kDa in mass, the permeability is restricted. The fibrillar structure on the nuclear side consists of eight fibrils attached to the nuclear ring of the core. The ends of the fibrils can interconnect, forming a basket. On the cytoplasmic side, there are eight fibril structures, making up an mRNP export interaction platform [242].

### Export kinetics

Transport of mRNPs through the NPC occurs in three steps: docking onto the nuclear basket, translocation through the central channel, and release from the cytoplasmic fibrillar structures. Measurements of fluorescently labelled single mRNPs regarding the time for NPC interaction vary, ranging from 12 ms to several seconds [9–12, 243, 244]. These differences may be due to technical differences or reflect variations for different mRNPs. In several cases, translocation through the central channel was fast, while times for docking and release were longer. Furthermore, only a minority (25–35%) of the interactions between the mRNPs and the NPCs resulted in export.

### Mechanism of translocation

At the ultrastructural level, the interaction between the NPC and endogenous gene-specific mRNPs has been best characterized for the BR mRNPs. The process lasts for 65 ms to several seconds [11]. In vivo, interactions between BR mRNPs with the NPCs result in productive translocations in a minority of the occasions. In 60–75% of the cases, the BR mRNPs return into the interchromatin compartment. The explanation for unproductive NPC association is not known, but may reflect that some mRNPs lack a full set of export receptors. In the case of BR mRNPs, about 75% of the mRNPs in the interchromatin lack the NXF1 receptor [12]. It is also possible that unprocessed mRNPs can be retained, as mediated by Mlp1 in yeast [245] and Tpr in mammals [231]. Since only a very small part of the pool of BR mRNPs in the interchromatin still contains introns (less than 5%) [111], we consider this explanation unlikely in this case.

When export occurs, a BR mRNP first interacts with the closed basket [246]. The basket ring opens up and the BR mRNP moves into the basket and subsequently into the opening of the central channel. This initial process involves Nup153 [247] and transient contact with Rae1 [248]. Several studies in other cell types demonstrate interactions between NXF1 and the basket component Tpr/Mlp1 at this stage. A complex between Rae1 and Nup98 is also involved [249].

For the BR mRNPs, a partial unfolding then follows and its bent 26 nm ribbon becomes more straight as it moves through the central channel. The 5′ end leads the way [250], while the 3′ end of the BR mRNP remains in contact with the basket ring. Other studies have shown that during translocation, NXF1 interacts sequentially with the FG Nups [251]. The transport of large mRNPs is facilitated if the cargo binds multiple export receptors. The exact mechanism of mRNP translocation is still unclear. Several transport models have been proposed for how export receptors (and import receptors) facilitate transport through the FG-Nup barrier. These include the selective phase, the reduction of dimensionality, and the virtual gating models [252, 253]. Extensive unfolding may be necessary for large mRNPs, such as the BR mRNPs. The reason for unfolding may be coupled to the translocation process or be dictated by space limitations of the NPC. We do not know if a conformational change is obligatory also for smaller mRNPs. Furthermore, the fact that BR mRNPs reach the cytoplasm with its 5′ end first could reflect efficient assembly of polysomes already in the perinuclear compartment, in a secretory cell. It remains to be demonstrated if this polarity of translocation is valid also for mRNPs, in general. If so, it could rather reflect the translocation process.

At the cytoplasmic side of the NPC, there is additional unfolding of the BR mRNP into a 7–10 nm mRNP fibre. Other studies have shown that upon exit from the central channel, mRNPs associate with the cytoplasmic part of the NPC [254]. A recent study has described how the Nups at the cytoplasmic side are oriented inward towards the central channel [242], presumably streamlining the passage of the mRNPs from the FG-repeats in the channel to a cytoplasmic platform. At this platform, the mRNPs are remodelled as a result of the coordinated action of the DEAD-box helicase DDX19 (in yeast Dbp5), Gle1 (in yeast Gle), and its cofactor inositol hexakisphosphate (IP6), and Nup214 (in yeast Nup159) [255]. Export factors are removed during remodelling, and eventually recycled back to the nucleus [256]. It is likely that many proteins remain bound to the mRNP. In the case of BR mRNPs, it has been demonstrated that, for example, SR, hnRNP, and Y-box proteins are not removed at this stage. The compositional and conformational change of the mRNP promotes directionality and it will prevent the mRNP from traveling back into the NPC. A deeper insight into the molecular changes in mRNP composition and where and when these changes occur in relation to NPCs and the translocation process will require high-resolution studies of endogenous mRNPs.

In the cytoplasm, the mRNP is again remodelled [257]. For example, the CBC proteins are replaced by eIF4E, thereby permitting steady-state translation and PABPN1 is replaced by PABPC, important for stability and translation.

## Formation and export of mRNPs linked to disease

Given the importance of mRNP formation and export for gene expression, it is not surprising that a great number of defects in these processes have been connected to diseases. An important field of investigation is how dysregulation of splicing can be involved in cancerogenesis [258, 259]. Splicing defects in cancer cells can result from base-pair substitutions in splice sites or in splicing regulatory sequences. Mutations can also affect the structure and function or expression levels of spliceosomal components and proteins that regulate splicing. Splicing defects have also been described in many other diseases [260]. These include neurological disorders such as Amyotrophic lateral sclerosis and Spinal muscular dystrophy.

Dysregulation of export components, including CRM1, THOC1, Rae1, and eIF4E, is described in different types of cancer cells [261]. In accordance with their roles in gene expression, several hnRNPs (hnRNP A1, C, and E1) influence expression of oncogenes in cancer cells and in neurons [262]. A possible link between mutations in a gene encoding a mammalian Nab2 orthologue and a neurodevelopment disorder has been reported [263]. Proteins connected to the NPC, for example, mammalian Nup88, Nup214, Nup98, and Gle1, have also been implicated in cancer, developmental, and neural diseases [264–266].

Replication of viruses depends on host cell gene expression machineries and can thereby affect mRNP formation and transport. Viruses are studied for the obvious medical implications for developing treatments against viral infections. A second reason is that viral infections often provide experimental advantages and short cuts in understanding cellular processes. This is exemplified by the fruitful studies of splicing of adenovirus pre-mRNAs [267].

During viral infection, the host cells activate defense mechanisms and the viruses interfere with the host cells to maximize viral replication. A common viral strategy is to shutoff host cell gene expression. In this way, the cellular immune response is evaded and resources are directed towards viral replication. Down-regulation of host cell gene expression can take place at most levels and individual viruses can use more than one mechanism.

Transcription can be inhibited. For example, the 3C protease of the poliovirus cleaves TFIID [268] and Sindbis virus protein nsP2 induces degradation of the largest subunit of RNA pol II [269].

Pre-mRNA processing is also targeted. The influenza virus protein NS1 binds CPSF and inhibits PABP binding, thus interfering with 3′ cleavage and polyadenylation [140, 270]. NS1 furthermore inhibits splicing by interfering with spliceosomal snRNP interactions [271]. The herpes simplex virus ICP27 protein inhibits splicing using multiple mechanisms [272, 273].

Many viruses, including herpes, influenza, and corona viruses, use viral or cellular endonucleases to degrade host cell mRNAs [274]. At the same time, it is important that viral mRNAs avoid being targeted for degradation [275].

Export of viral mRNAs from the nucleus to the cytoplasm has attracted considerable interest. In general, viruses take over cellular export factors, for example, the NXF1 and CRM1 export receptors, using a variety of strategies. Several viruses produce mRNAs that contain elements that can bind TREX [276] or that can directly bind NXF1 [277]. Other viruses, such as influenza and Hepatitis B viruses, produce specific export adaptor proteins. These proteins recruit ALY/REF or DDX39B and TREX and subsequently NXF1 [278, 279]. Other examples of cis-acting elements present in viral mRNAs that can bind export factors are the element in type D retroviruses that directly bind NXF1 [277, 280], and the Rev response element in HIV-1 mRNA that binds multimerized Rev proteins which, in turn, bind CRM1 [281, 282].

## Future perspectives

A deeper understanding of the processes of gene expression in the intact cell nucleus will have a great impact on many aspects of biology, ranging from evolution to cell differentiation and neurobiology. It will also be essential for diagnosis and treatment in most disciplines of medicine. It is important to continue to identify and characterize processes and the involved individual components, probably with an emphasis on multi-molecular complexes and the dynamic changes of such complexes. The most important and, at the same time, most challenging area is how everything operates in the living cell. Knowledge about the compositional and conformational dynamics of complexes and their interactions must be studied in the perspective of functional importance for endogenous genes in intact cells. This goal is demanding and exciting, and will require that new methods are developed. Such methods should be able to map the interplay within and between RNA–protein and protein–protein complexes, including the temporal and spatial dimensions. As an example, it is already evident that the pre-mRNP, the processing machineries, and the chromatin are all in very close contact. It is desirable to learn which interactions are the functionally important ones. Furthermore, we need to know more about how various components are recruited to sites of action and thereafter recycled, in the intact cell.
